# Four Genotyping Schemes for Phylogenetic Analysis of *Pseudomonas aeruginosa*: Comparison of Their Congruence with Multi-Locus Sequence Typing

**DOI:** 10.1371/journal.pone.0082069

**Published:** 2013-12-11

**Authors:** Makaoui Maâtallah, Amina Bakhrouf, Muhammed Asif Habeeb, Agata Turlej-Rogacka, Aina Iversen, Christine Pourcel, Olfa Sioud, Christian G. Giske

**Affiliations:** 1 Laboratoire d'Analyse, Traitement et Valorisation des Polluants de l'Environnement et des Produits, Faculté de Pharmacie, Monastir, Tunisia; 2 Clinical Microbiology L2:02, MTC – Karolinska Institutet, Karolinska University Hospital Solna, Stockholm, Sweden; 3 Univ Paris-Sud, Institut de Génétique et Microbiologie, Orsay, France; 4 CNRS, Orsay, France; 5 Laboratoire de Microbiologie CHU Fattouma Bourguiba, Monastir, Tunisia; The University of Hong Kong, China

## Abstract

Several molecular typing schemes have been proposed to differentiate among isolates and clonal groups, and hence establish epidemiological or phylogenetic links. It has been widely accepted that multi-locus sequence typing (MLST) is the gold standard for phylogenetic typing/long-term epidemiological surveillance, but other recently described methods may be easier to carry out, especially in settings with limited access to DNA sequencing. Comparing the performance of such techniques to MLST is therefore of relevance. A study was therefore carried out with a collection of *P. aeruginosa* strains (n = 133) typed by four typing schemes: MLST, multiple-locus variable number tandem repeat analysis (MLVA), pulsed-field gel electrophoresis (PFGE) and the commercial DiversiLab microbial typing system (DL). The aim of this study was to compare the results of each typing method with MLST. The Simpson's indices of diversity were 0.989, 0.980, 0.961 and 0.906 respectively for PFGE, MLVA, DL and MLST. The congruence between techniques was measured by the adjusted Wallace index (W): this coefficient indicates the probability that a pair of isolates which is assigned to the same type by one typing method is also typed as identical by the other. In this context, the congruence between techniques was recorded as follow: MLVA-type to predict MLST-type (93%), PFGE to MLST (92%), DL to MLST (64.2%), PFGE to MLVA (63.5%) and PFGE to DL (61.7%). Conversely, for all above combinations, prediction was very poor. The congruence was increased at the clonal complex (CC) level. MLST is regarded the gold standard for phylogenetic classification of bacteria, but is rather laborious to carry out in many settings. Our data suggest that MLVA can predict the MLST-type with high accuracy, and even higher when studying the clonal complex level. Of the studied three techniques MLVA was therefore the best surrogate method to predict MLST.

## Introduction

The metabolically versatile gram-negative bacterium *Pseudomonas aeruginosa* has an extraordinary ability to adapt and colonize several ecological niches [1]–[5]. This opportunistic pathogen has dispersed globally, causes a variety of infections, and is frequently acquired in hospital environment, such as intensive care units (ICU) [6], [7]. Because it represents a major health burden in hospitalized patients, and due to its scientific and medical interests, several studies have investigated the dissemination of virulent or drug-resistant clones.

The analysis of bacterial pathogens by various typing methods provides important information for establishing the genetic relatedness among isolates for the purposes of long-term epidemiological studies and/or outbreak investigations [8], [9]. Hence, strain typing has two major aims: (i) to index genetic microvariation for use in outbreak investigations and (ii) to index genetic macrovariation for use in phylogenetic and population-based analyses [10]. Generally, phylogenetic methods were developed to elucidate evolutionary history of strains and depicting their patterns of relatedness. An accurate phylogeny attempts to unravel the crossed wires of evolutionary relationships among samples of populations and elucidates how they are related and diverged from each others. In addition, it provides the ability to predict the phenotypic and genotypic traits, permitting a better understanding of the ecology and the dynamic of the bacterial population biology [11], [12]. Phylogenetic analyses are often conducted to determine whether one particular outbreak may be related to another during times of an epidemic [13], and whether certain strains are more often associated with outbreaks than others [14].

For over 20 years, genome fingerprinting was a tool of pivotal importance to track the transmission routes of microbial pathogens. It is a highly useful strategy for surveillance and outbreak investigation of human infections and evolutionary analysis [15], [16]. Several molecular techniques have been successfully applied to differentiate bacterial strains and clonal groups [17]. The major success factor of a typing system lies in the discriminatory power, i.e. the ability to distinguish between epidemiologically related and unrelated strains [8]. Each technique has its characteristics and its applicability and the limitation of such techniques can be discerned. In some cases the inference of relationship among isolates by one typing method does not correlate with that obtained by another [18].

Typing methods have evolved from phenotypic methods to genotypic methods. Several schemes have been adapted to type *P. aeruginosa* associated to cystic fibrosis patients and isolates featuring antibiotic resistance determinants. The traditional phenotypic markers are known to be unstable and do not offer satisfactory resolution power for discrimination of strains. A wide variety of molecular genetics methods were generally introduced to fulfill those shortcomings. Some of them were PCR-based methods such as Random Amplified of Polymorphic DNA (RAPD)[19] and rep-PCR [20]. Other techniques were based on Restriction Endonuclease Analysis (REA) of the total genome such as Pulsed-Field Gel Electrophoresis (PFGE) [21], or partial fragments of the genome such as ribotyping [22]–[24]. PFGE has been regarded the ‘gold standard’ molecular typing method for a variety of bacterial species for outbreak investigation purposes [25]. Another technique, Amplified Fragments-Length Polymorphism (AFLP) has been shown in some studies to offer a discriminatory power comparable to that of PFGE [26]. Technically it can provide results more rapidly than PFGE [27], but it has several limitations in terms of being challenging to standardize, labor intensive, and also relatively subjective [25].

Given the need to validate new portable and standardizable approaches, Multi-Locus Sequence Typing (MLST) [28] schemes, were developed, which are capable of measuring the genetic variation in house-keeping genes. MLST is fully standardized for numerous bacterial species [29], and is able to detect phylogenetically informative genetic variations retrieved from the strictly conserved studied genes. Therefore, this scheme is capable of differentiation between strains, and of accurately tracking the global clonal history of different species. For several pathogens MLST is a preferable tool, especially when epidemiological, geographical and/or evolutionary studies need to be carried out [30]–[32]. Another relatively new technique, Multilocus Variable Number Tandem Repeat Analysis (MLVA), has been successfully used for epidemiologically studies for several species. Onteniente et al. developed the first MLVA scheme for *P. aeruginosa* which consisted of seven VNTR markers [33]. Later, the MLVA scheme (MLVA-15) was updated by improving the protocol and adding new discriminative markers, making the assay more robust and discriminatory. The MLVA-15 scheme is thus assumed to be highly informative and useful for epidemiological surveillance of *P. aeruginosa* infections [34]. Moreover, MLVA has become a useful tool for outbreak detection and source tracing in European countries [35]. Interestingly, it has been reported that an accurate phylogeny could be established from an MLVA scheme containing several VNTR loci displaying a wide range of diversity [36], [37].

More recently, a new rep-PCR-based technique was introduced: the DiversiLab system (DL, bioMérieux, Marcy l'Etoile, France). This system gives more reproducible results than standard rep-PCR approaches, as the analysis is fully automated. The new system has been evaluated for its usefulness in determining hospital outbreaks with commonly occurring pathogens. Few reports have so far evaluated the performance of this system for *P. aeruginosa* typing, and there is no clear consensus regarding the performance and usefulness of this scheme [38]–[41]. In parallel, it has been suggested that DL can be used for phylogenetic purposes in *Klebsiella pneumoniae* [42], [43].

In a previous study we used PFGE and MLST to investigate the population structure of *P. aeruginosa* isolated from five Mediterranean countries [29]. From this study we selected a representative collection consisting of 133 isolates, which was subjected to typing with MLVA and DiversiLab. The primary aim of this study was to analyze the genetic heterogeneity of *P. aeruginosa* by each technique and compare their discriminative power and congruence, especially with MLST sequence types or clonal complexes, as these are regarded most predictive of long-term epidemiological relationships.

## Materials and Methods

### Bacterial strains

A representative collection of strains from five Mediterranean countries (Tunisia, Libya, France, Spain and Italy), previously typed by MLST and PFGE [29] were used in the present study. Furthermore, four metallo-β-lactamase (MBL)-producing strains having distinct STs: AK5493 (ST229; Sweden), VR143 (ST227; Italy), 134MG (ST228 Italy) and PA66 (ST230; Sweden), were used. Overall, the bacterial collection consisted of isolates from various geographical origins and derived from several backgrounds of infections. The collection included several important clones such as the CC235 clone and two isolates (environmental and clinical) belonging to the most successful clone C. The latter has shown a remarkable spread in European countries in both the environment and cystic fibrosis patients [44]. Three reference strains (ATCC 27853, PAO1 and PA14) were also included. These strains served for the calibration of the experiment. ATCC 27853 was used as a control for both PFGE and DiversiLab, whereas PAO1 and PA14 were used as control strains for MLVA.

### PFGE and MLST

PFGE typing was performed as previously described [45] with minor modifications. In short, all isolates were digested with the *Spe*I restriction enzyme (New England Biolabs, Hirts, United Kingdom). DNA fragments were separated in 1.2% agarose (SeaKem® Gold Agarose) in a CHEF-Mapper (Bio-Rad, Hercules, USA) in 0.5X Tris-Borate EDTA (TBE) running buffer at 12°C and 6 V/cm for 30 hours with pulse time ranging from 1 to 50 s. *P. aeruginosa* ATCC 27853 was used as a reference and included in every 6 lanes to allow normalization of gels. Gels were stained with ethidium bromide, photographed and saved as a TIFF files in Geldoc EQ (BioRad Laboratories, Hercules, CA). The resulting photographic images were analyzed with the GelCompar II software (Applied Maths, NV St-Martens-Latem, Belgium). The band patterns were compared using the Dice-coefficient by using the Unweighted Pair Group Method with Arithmetic Mean (UPGMA) to determine band similarity accordingly to the criteria established by Tenover et al. [46]. Isolates producing PFGE fingerprints with ≤6 bands differences and with ≥80% similarity were categorized as the same PFGE type (PT).

MLST was performed according to the protocol of Curran et al. [28] with minor adjustments using the newly designed primers [29]. Briefly, for DNA extraction, overnight cultured *P. aeruginosa* isolates were heated to 100°C for 10 min. The seven housekeeping genes (*acsA*, *aroE*, *guaA*, *mutL*, *nuoD*, *ppsA* and *trpE*) were amplified for all isolates by real-time PCR using Rotorgene 6000 (Corbett Robotics Inc; San Francisco, CA, USA). The PCR reaction was carried out using the QuantiTect SYBR Green PCR mix (Qiagen, Valencia, CA, USA). The amplified products were sequenced on both strands with the published primers and the newly designed primers using the BigDye Terminator Ready Reaction Mix v3.1. Nucleotide sequences were determined for both strands by ABI Prism 3100 Genetic Analyzer (Applied Biosystems, Foster City, CA). The results were further analyzed with the BioNumerics7 software (Applied Maths, St-Martens-Latem, Belgium) in order to assign the allelic numbers and sequence types (STs). The obtained results were compared with the available alleles in the MLST database (http://pubmlst.org/paeruginosa).

In addition, the BioNumerics software was used to manage the data and perform the clustering analysis. The minimum spanning tree (MST) is an alternative phylogenetic network generated by an algorithm implemented in BioNumerics and created for both MLST and PFGE data. MLST allelic profiles were used as categorical data. Isolates with five or more identical alleles were considered as part of the same clonal complexes (CCs). Concerning PFGE, an MST with permutation resampling was generated based on the band matching class table which served as input data. The available collapsing tool was used to define the closely related isolates with ≤6 band class differences.

### MLVA

The annotated genome sequences of the reference strains PAO1 and PA14 were scanned and inspected for the presence of potential VNTR loci by using the strain comparison tool developed by Denoeud and Vergnaud [47], available at the Microbial Tandem Repeats Database (http://minisatellites.u-psud.fr/).

Bacterial thermolysates were used as source of DNA. For MLVA typing, the PCR reaction was performed using the published primers adapted from the protocol by Vu-Thien et al. [34]. These authors designed a new MLVA scheme, based on a 15 candidate VNTR loci grouped into two panels: panel 1 with 13 minisatellite loci (ms77, ms127, ms142, ms172, ms211, ms212, ms213, ms214, ms215, ms216, ms217, ms222 and ms223) and panel 2 with 2 microsatellite loci (207 and ms209). The amplification reactions were carried out using PTC 200 thermocycler (MJ Research Inc., Ramsey, MN, USA), as follows: initial denaturation step at 94°C for 5 min, 35 cycles (denaturation for 30 s at 94°C, annealing for 30 s at 60°C, and extension for 45 s at 72°C), followed by a final extension step at 72°C for 10 min.

For the minisatellites, PCR products were separated in 2% in Nusieve® 3:1 Agarose using GNA-200 machine characterized by a 20-cm-wide gels made in 0.5X TBE buffer and run at 8 V/cm. The amplified PCR products of the reference strains (PAO1 and PA14) and 100 bp marker (Fermentas GmBH, St. Leon-Rot, Germany) were run on each gel. The same procedure was used for microsatellites, with some modifications. PCR products were separated in a 4% agarose gel made with 2% of Nusieve® 3:1 Agarose plus 2% MetaPhor® Agarose. A 20 bp ladder size marker (Fermentas GmBH, St. Leon-Rot, Germany) was used to analyze the 6 bp repeat units of ms207 ms and ms209 markers. Uncertain band sizes and unexpected PCR products were subjected to DNA sequencing to improve the analysis and facilitate the interpretation. The size of each amplicon was deduced by visual inspection and measured using BioNumerics and the number of repeats was deduced using the MLVA alleles assignment table on the genotyping site (http://minisatellites.u-psud.fr/MLVAnet/). The number of repeats in the alleles was estimated by subtracting the invariable flanking region from the amplicon size divided by the repeat unit length, as determined for reference strains PAO1 and PA14 and according to the formula: number of repeats (bp)  =  fragment size (bp) - flanking regions (bp)/repeat size (bp). Each locus was given an allele number and each isolate generated an allelic profile consisting of a string of 15 numbers reflecting the number of repeats in the 15 VNTR loci. The combination of all VNTR repeats generated an MLVA-type (MT). A distinct MT was assigned on the basis of at least one differing number. All profiles among which new MTs were discovered in this study were deposited in the database. The polymorphism index for individual VNTR loci was expressed as the Hunter-Gaston diversity index (HGDI), an application of Simpson's diversity index (SID) [48].

The BioNumerics software package was used to perform clustering using the categorical coefficient to generate dendrograms based on the UPGMA method or to draw an MST. The latter method is a graphical tool illustrating the total members of the population and allowing visualization of all genotypes into one single compact image. The same setting adopted for both MLST and MLVA data is that an MST was created based on the categorical coefficient and a priority rule consisting of the highest number of single-locus variants (SLVs). For MLVA, groups or clonal complexes were created if 13 out of the 15 MLST loci were identical [34].

### DiversiLab (DL) system (rep-PCR)

*P. aeruginosa* DNA was extracted using the UltraClean™ Microbial DNA Isolation Kit (Mo Bio Laboratories Inc., Solana Beach, CA) according to the manufacturer's instructions. The DNA quantification was measured using the NanoDrop ND-1000 spectrophotometer (NanoDrop Technologies, Wilmington, DE, USA). Then, rep-PCR was performed using PTC 200 thermocycler and Pseudomonas fingerprinting kit (Bacterial Barcodes, bioMérieux, Athens, GA, USA) in a total reaction volume of 25 µl. The reaction mixture consisted of 18 µl of rep-PCR MM1, 2.5 µl of Gene Amp 10X, 2 µl of primer Mix, 0.5 µl of AmpliTaq DNA polymerase (Applied Biosystems, Foster City, CA, USA) and 2 µl of genomic DNA (25-50 ng/µl).

Thermal condition was as follows: initial denaturation step at 94°C for 2 min, 35 cycles (denaturation at 94°C for 30 s, annealing at 50°C for 30 s, extension at 70°C for 90 s), followed by a final extension step at 70°C for 3 min. The PCR products were analyzed using the Agilent 2100 BioAnalyzer (Agilent Technologies, Santa Clara, CA, USA). Then, the amplified fragments (sizes from 100–1000 bp) were electrophoretically separated with the microfiluidic labchip. In order to monitor reproducibility, the reference strain of *P. aeruginosa* ATCC 27853 was used as a control in each PCR reaction and each chip. Electropherograms were downloaded and automatically analyzed by the DiversiLab® (version 3.4). All fingerprint patterns were normalized, then, the Pearson correlation coefficient was used in order to calculate the distance matrices among all samples. Based on the UPGMA and the multidimensional scaling, the DiversiLab® software created a customized report presenting a dendrogram, electropherograms, virtual gel images and scatter plots. The relatedness among isolates was deduced as previously described [39]: linked isolates (similarity above 95%) and different (similarity less than 95%). An alternative cut-off (similarity index: 93%) was tested to evaluate whether it had a better correspondence to MLST findings. The DiversiLab data were visualized in BioNumerics as an MST with permutation resampling, showing all genotypes into one graph. Closely related isolates differing by a maximum of two band classes were collapsed into one node.

### Index of diversity and concordance of techniques

The index of diversity and the degree of congruence among different typing schemes were determined via an online tool (http://www.comparingpartitions.info/; accessed on 23/01/2013). The discriminatory ability of the above described techniques was evaluated using the Simpson's index of Diversity (SID: with 95% confidence intervals) as described by Hunter and Gaston [48]. An index greater than 0.90 is considered desirable if the typing results are to be interpreted with confidence [48]. Inter-method concordance was calculated using the adjusted Rand and Wallace coefficients; the adjusted Rand index (R) shows the proportion of agreement corrected for the presence of chance agreement whereas the Wallace coefficient (W) indicates the probability that two strains classified as the same type by one method will also be classified as the same one when using the other method [49].

## Results

### PFGE

**Figure 1:**
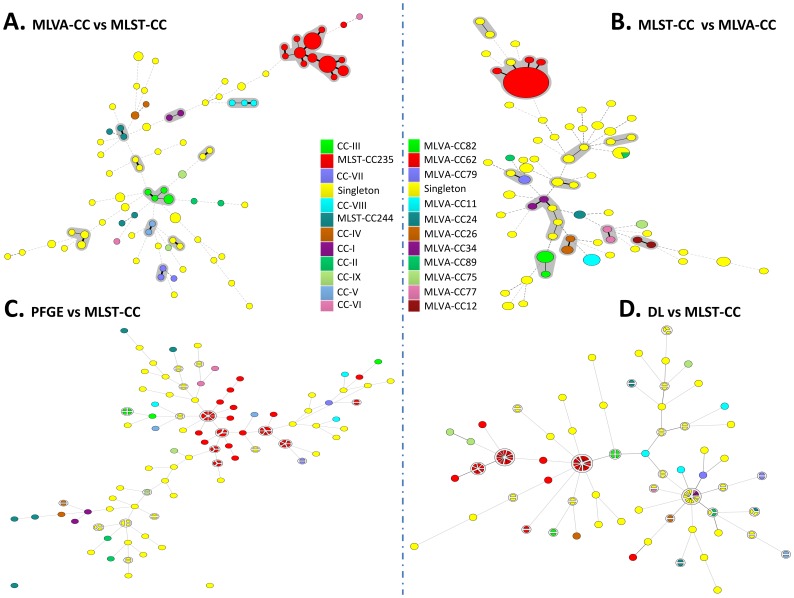
Minimum Spanning trees (MSTs) of 133 *P. aeruginosa* isolates based on MLVA, MLST, PFGE and DiversiLab data. Each network represents its own genetic relatedness among isolates and displays its relative concordance against MLVA-CC and MLST-CC. A; Clustering of MLVA profile was done using the categorical coefficient. Each circle represents an MT, the size of which indicates the number of isolates with this particular type. Black lines connecting pairs of MTs indicate that they differ in one VNTR locus (thick lines), two VNTR locus (thin), or three to 15 VNTR locus (dashed). Grey zones surround MTs that belong to the same MLVA-CC. B; Clustering was done using MLST character data. Each circle represents an ST, the size of each circle indicates the number of isolates with this type. Black lines connecting pairs of STs indicate that they differ in one allele locus (thick lines), two alleles locus (thin), or three to seven (dashed). Grey zones surround STs that belong to the same MLST-CC. C; MST with permutation resampling with majority summary based on band matching class, each single node represents a distinct PFGE-type, the collapsed nodes represent closely related patterns with ≤6 band class differences. D; MST with permutation resampling with majority summary based on band matching class, each single node represents a distinct DL pattern, the collapsed nodes represent closely related patterns with ≤2 band class differences.

Using a similarity cut-off of 80%, the 133 isolates produced 90 PFGE-types, designated PFGE 1 to 90. In addition, the results revealed twenty clonal groups consisting of two or more isolates, representing closely related patterns (Table 1). Group 17 was the largest with 8 isolates. The remaining 70 unique PFGE-types were categorized as singletons (Fig. 1C, Table S1).

**Table 1:** Number of genotypes, clonal complexes and singletons retrieved from the four genotyping schemes.

Typing methods	No. of types	SID	95% CI
**PFGE**	90	0.989	(0.984–0.995)
**MLVA**	85	0.980	(0.970–0.991)
**DL (cut-off 95%)**	61	0.961	(0.946–0.977)
**DL (cut-off 93%)**	46	0.937	(0.915–0.958)
**MLST**	67	0.906	(0.860–0.951)

### MLST

**Figure 2:**
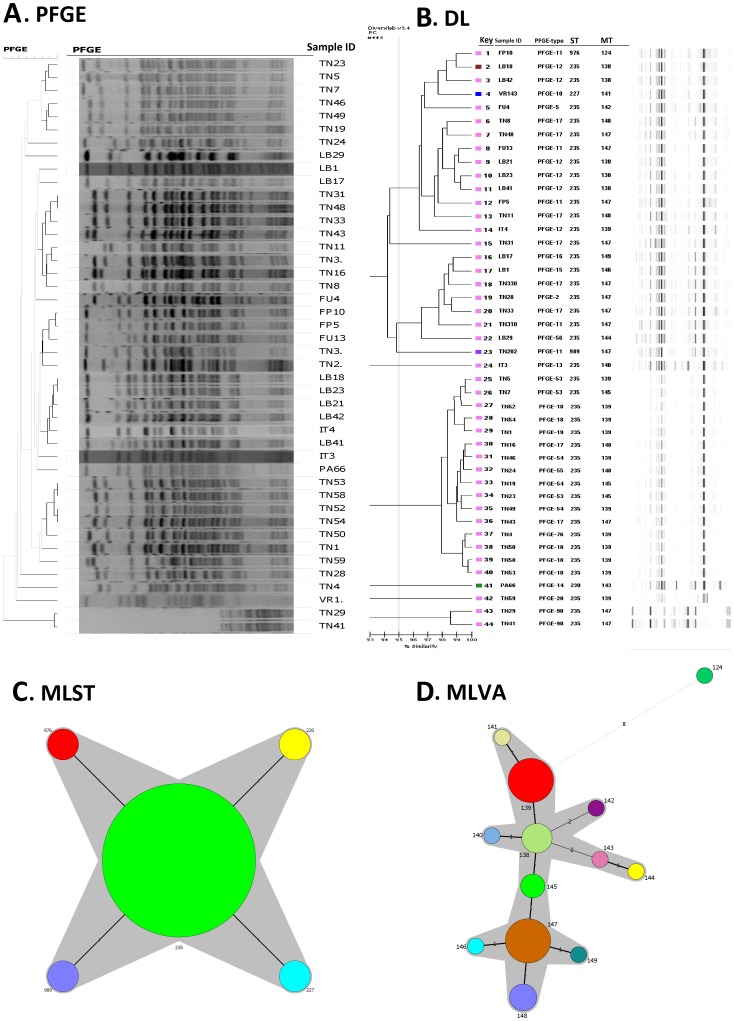
Graphical representation of 44 isolates belonging to MLST-CC235 analyzed by PFGE, DL, MLST and MLVA. A; Dendrogram generated by PFGE banding pattern, the clustering was done by UPGMA using the Dice coefficient with a tolerance position of 1%. 19 PFGE types were obtained with a cut-off of 80% of similarity. B; Dendrogram generated by the Pearson cluster analysis of rep-PCR results. PFGE-type, ST, MT and virtual gel are shown for each isolate. STs are depicted in colored boxes. A similarity index cut-off value of 95% was used by the DiversiLab® software to define genetic classification and to highlight the 9 DL-types. C; Minimum Spanning Tree illustrating the clonal complex MLST-CC235, the predominant ST235 is the primary founder surrounded by its SLVs (STs 976, 230, 989 and 227). Each color was assigned to each distinct ST. D; Minimum Spanning Tree of MLST-CC235 isolates typed by MLVA, Each circle represents an MT and 13 different colors were assigned to each distinct MT.

MLST was performed for all strains. Sixty-seven STs were assigned and segregated into 11 clonal complexes (Fig. 1B, Table 1). The main clonal complex was MLST-CC235 (Fig. 2C), consisting of five STs (235, 989, 979, 230 and 227, with ST235 as the primary founder) followed by the clonal complex CC244 with five STs (244, 990, 986, 993 and 654 with ST244 as the primary founder). The remaining groups were doublets or had a simple association among couples of STs (Fig. 1B, Table S1). Thirty-nine unrelated STs were considered as singletons.

### MLVA

With some exceptions (n = 17) that did not generate any band for a few markers, all isolates were successfully typed. For 8 isolates, unexpected bands above 1500 bp were observed. This is probably due to the presence of an insertion sequence (IS) [34] in the VNTR especially for ms214. MLVA-15 was able to subdivide the studied population into 85 MTs (Fig. 1A, Table 1, Table S1). Most of them were new MTs (n = 70). As shown in the MST, the resulting MTs were segregated into 11 MLVA clonal complexes. MLVA-CC62 was the largest lineage, consisting of 12 MTs (Fig. 1A, Fig. 2D), and was shared by 43 isolates belonging to MLST-CC235. The remaining 10 MLVA-CC (Fig. 1A) were as follows: MLVA-CC79 (70, 71), MLVA-CC82 (76, 77, 78 and 79), MLVA-CC12 (17 and 99), MLVA-CC75 (103 and 104), MLVACC-34 (90 and 91), MLVA-CC24 (101 and 102), MLVA-CC26 (39, 108 and 109), MLVA-CC11 (15, 112 and 113), MLVA-CC77 (96 and 97) and MLVA-CC89 (86 and 87).

### DiversiLab

All isolates were typed by the automated rep-PCR-based DiversiLab system. By using a similarity cut-off of 95%, DL typing differentiated the 133 isolates into 61 DL-types, whereof 36 were singletons or unique patterns (Table 1). The remaining DL groups represented the clonally related isolates comprising two or more isolates. DL 1, 10 and 27 (arbitrary numbers) were the largest groups, comprising 14, 13 and 16 isolates, respectively (Fig. 1D, Table S1). Interestingly, strains belonging to DL 1 and 27 belonged to MLST-CC235 and MLVA-CC62, respectively. The alternative cut-off of 93% decreased the total cluster by subdividing the whole population into 46 clusters.

### Simpson index of diversity (SID) and concordance between typing schemes expressed by the Wallace coefficient (W)

The SID of all the studied techniques was above 0.9 (Table 2) indicating a relatively high level of discrimination of all techniques. As suspected, PFGE had the greatest index (0.989; cut-off ≥80%), followed by MLVA (0.980), DL (0.961/0.937; cut-off 95/93%) and MLST (0.906).

**Table 2:** Simpson's index of diversity of the different typing methods.

Typing methods	MLST	MLVA	PFGE	DiversiLab
**Genotypes/types**	67 STs	85 MTs	90 PFGE-types	61 DL-types
**Clonal complexes/clonal groups**	11	11	20	25
**Singletons/unique patterns**	39	49	70	36

SID, Simpson's index of diversity.

CI, confidence interval.

The clustering analysis of the studied collection by all typing schemes revealed a lot of related and distinct genotypes but not totally overlapping from one technique to another. The adjusted Wallace coefficient (Table 3) indicated that MLST-type at the ST-level was well predicted by MLVA (0.930) and PFGE (0.919), but less well predicted with DL (0.642/0.562; cut-off 95/93%). Conversely, MLST, as expected, was not able to predict types retrieved using any of the other techniques. MLVA-type was not predicted by the other techniques except to some extent with PFGE (0.635). DL-type was predicted better by PFGE (0.617/0.786; cut-off 95/93%) than other techniques. PFGE-type could not be predicted by any of the other techniques.

**Table 3:** Congruence between typing methods determined by the Adjusted Wallace coefficient [95% CI].

Typing methods	MLST	MLST-CC	MLVA	MLVA-CC	DL (cut-off 95%)	PFGE (cut-off 80%)	DL (cut-off 93%)
**MLST**		1.000 (1.000–1.000)	0.181 (0.111–0.251)	0.993 (0.987–1.000)	0.250 (0.167–0.333)	0.097 (0.052–0.141)	0.366 (0.274–0.458)
**MLST-CC**	0.791 (0.633–0.949)		0.154 (0.089–0.219)	0.928 (0.839–1.000)	0.229 (0.152–0.305)	0.083 (0.045–0.121)	0.337 (0.240–0.434)
**MLVA**	0.930 (0.818–1.000)	1.000 (1.000–1.000)		1.000 (1.000–1.000)	0.486 (0.342–0.629)	0.343 (0.240–0.447)	0.601 (0.418–0.784)
**MLVA-CC**	0.845 (0.698–0.993)	0.999 (0.998–1.000)	0.165 (0.098–0.233)		0.229 (0.150–0.309)	0.085 (0.046–0.125)	0.335 (0.238–0.433)
**DL (cut-off 95%)**	0.642 (0.547–0.737)	0.742 (0.706–0.778)	0.242 (0.137–0.347)	0.692 (0.612–0.771)		0.166 (0.096–0.237)	0.959 (0.898–1.000)
**PFGE (cut-off 80%)**	0.919 (0.868–0.969)	1.000 (1.000–1.000)	0.635 (0.511–0.758)	0.953 (0.897–1.000)	0.617 (0.530–0.704)		0.786 (0.662–0.911)
**DL (cut-off 93%)**	0.562 (0.461–0.663)	0.655 (0.628–0.682)	0.179 (0.100–0.259)	0.605 (0.525–0.685)	0.574 (0.472–0.676)	0.127 (0.073–0.181)	

CI, confidence interval.

MLST-CC, MLST clonal complex.

MLVA-CC, MLVA clonal complex.

We found high bidirectional agreement between MLST-CC and MLVA-CC (Table 3, Fig. 1A, B). However, neither MLST-CC nor MLVA-CC could predict PFGE and DL (Table 3). PFGE predicted the MLST-CC-type at 100% level (Fig. 1C, Table 3), whereas the DL predicts the MLST-CC-type at a 74/65%; cut-off 95/93%) level (Fig. 1D, Table 3). When analyzing separately the 44 MLST-CC235 isolates, they were split into 19 PFGE-types (SID = 0.926) (Fig. 2A), 13 MTs (SID = 0.840) (Fig. 2D), 9 DL-types (SID = 0.754) (Fig. 2B) and 5 STs (SID = 0.175) (Fig. 2C). All these isolates belong to the MLVA-CC62 except the isolate FP10, which had a divergent MLVA profile (Fig. 2D).

## Discussion

The population biology of *P. aeruginosa* has been extensively investigated. There is a consensus that the population structure can be defined as a non-clonal, including several heterogeneous clones but punctuated by some major epidemic clones or clonal complexes [28], [29], [50]–[52]. This study was carried out using a set of isolates collected from several Mediterranean countries. The strains were characterized and typed by four molecular typing schemes (MLST, PFGE, MLVA and DL).

### Assessment and nature of genetic variations retrieved by the four genotyping schemes

The selected isolates were all typeable by the selected genotyping schemes. PFGE showed a high index of diversity (SID = 0.989), as discussed in other studies [53], [54]. The amount of PFGE variation is due to genetic events that alter the size of the restriction fragments, such as modification of restriction sites by point mutations or insertions/deletions of longer DNA sequences. The MLST variation is restricted to changes within housekeeping genes, while MLVA variation is restricted to the tandem repeats on the selected VNTR loci. The DL variation is mediated by non-coding repetitive sequences dispersed along the genomes. The high resolution power of PFGE makes the method well suited for outbreak investigations, but less well suited for tracking long-term epidemiological relations [26], [55], [56].

MLVA-15 subdivided the population into 85 genotypes or MTs indicating a high level of discrimination (SID = 0.98). The available MLVA profiles in the database are skewed towards clinical isolates from cystic fibrosis patients, or MDR isolates from patients of other categories [33], [34], [54], [57]–[60]. Even though these studies use different MLVA schemes, they have pointed out the usefulness and discrimination of the MLVA protocol for *P. aeruginosa* genotyping. More recently, a new automated protocol of MLVA, named MLVA-16 which is assayed in two multiplexed PCR, was developed [61]. The MLVA-16 contains all the MLVA-15 VNTR loci [34], [59] and an additional ms61, which is a highly polymorphic loci, previously used in the MLVA-9 [57]. Importantly, this additional microsatellite increases the discrimination of the method by adding further insight into the strain evolution during long-term colonization. Remarkably, one interesting feature of MLVA is that the index of diversity can be increased to a great extent by including new VNTR markers. The findings with the MLVA-15 used in our study are in line with earlier observations by displaying diversity values ranging from 50 to 88% (Table S2). The VNTR loci displaying lower or moderate diversity are useful for establishing deeper phylogenetic relationships and for the definition of clonal complexes, whereas markers with higher level of diversity offer a greater discriminatory power among closely related isolates [62].

DiversiLab was found to have a relatively high resolution power, separating isolates into 61 DL-types with an index of diversity of 0.961, at the 95% similarity cut-off level. When using the relaxed cut-off at 93% similarity, the correlation with MLST decreased. Few reports have evaluated the utility of DiversiLab system, however, some have found an equal capability to that of PFGE for the confirmation of the circulation of multidrug-resistant *P. aeruginosa* clones in different hospital wards [38]. Other studies have typed a collection of *P. aeruginosa* obtained from different epidemiological situations and provided results similar to those obtained with PFGE. [39]. According to our findings, DL could identify closely related isolates identified by PFGE or MLST in some cases. On the other hand, it overestimated the relatedness since some isolates belonging to the same DL groups were differentiated by PFGE into several types. Other isolates belonging to the same MLST-CC or PFGE group were grouped differently based on DL. The DiversiLab system has been applied for many bacterial species, it was evaluated for its performance and feasibility for the identification of hospital outbreak, and it has shown to be useful for some species and less for others like *P. aeruginosa* [40].

MLST subdivided the population into 67 STs with an index of diversity of 0.906. Although MLST has limitations in terms of resolution power, the method is now clearly considered the gold standard for phylogenetic studies. The method identifies SNPs as well as genomic rearrangements located in the seven conserved genes, allowing phylogenetic analysis by tracing the evolutionary history between strains and identifying a subset of isolates genetically appearing related through a common ancestor. The main drawback of MLST is that the method is time-consuming and costly. In order to overcome these limitations and given the importance of the scheme, Boers et al. [63] developed an MLST protocol for *P. aeruginosa* and other species using next-generation sequencing (NGS). The high throughput MLST (HiMLST) protocol consisted of two standardised steps increasing the typing potential of MLST and promising a high quality and cost effective typing of bacterial strains. In parallel, in order to speed up the current MLST protocols, new microfluidic platform technologies are currently developed [64].

### Congruence of the three schemes with MLST at the ST and clonal complex levels

The adjusted Wallace coefficient (Table 3) indicates the probability that pairs of isolates which are assigned to the same type by one typing method are also typed as identical by the other. MLST-types were best predicted by MLVA, followed by PFGE and DL. By contrast MLST is not a good method to predict MLVA-, PFGE- and DL-types. The discrepancies were mainly observed in some isolates having the same STs, but dispersed into different PFGE groups, MLVA-types or DL groups. PFGE has a good unidirectional prediction to MLST, MLVA and DL, but due to the higher resolution of PFGE other methods perform poorly in terms of predicting PFGE-types.

Regarding MLVA, all MLST-CC235 isolates except one belonged to the same MLVA-CC62. Our findings confirm the high level of agreement between MLST and MLVA at the clonal complex level. Likewise the bidirectional congruence between MLST-CC and MLVA-CC was well supported by the Wallace coefficient in (Table 3) and well visualized in MSTs in (Fig. 1A, B). On the other hand, the congruence was univocally increased among PFGE to MLST-CC (W = 100%), DL to MLST (W = 74%).

As seen in Table 3, the unidirectional congruence of techniques to others and absence or limited congruence of the counterpart is difficult to refer to one factor but could occur for several reasons. A large amount of polymorphisms was detected and the phylogenetic incongruence is mainly due to the significant amount of homoplasy, homologous recombination and lateral gene transfer [13], [65], [66]. However, it is difficult to find optimal markers establishing a real phylogenetic history. Ideally, SNPs which are relatively rare and scattered through the genome are more evolutionary informative than any other markers. These SNPs display low mutation rates, and can provide a relatively high level of phylogenetic resolution [13], [67]–[72]. Interestingly, the genetic data provided by the fingerprint-based methods such as PFGE and DL could somehow predict the clonal level, but provides no specific DNA sequence information. The rep-PCR relies on short repeated stretches of DNA abundant in eubacterial genomes. The PFGE strategy makes uses of rare cutting sites dispersed in the whole genome. The DNA information provided by both approaches is therefore clearly affected by recombinational events [39]. Hence, they are useful for identifying closely related isolates in short time scale but cannot be used for understanding the underlying genetic diversity and evolutionary history of population isolates. Generally, for populations such us *P. aeruginosa* undergoing significant recombination, phylogenies inferred by the PFGE [9] or DL data are inaccurate and therefore misleading. MLVA can enhance the resolution within epidemiologically related isolates clustered by MLST. Currently the genotyping by MLVA is in progress, and more investigation is needed to fully appraise the potential of this approach as a typing tool. MLVA allows the differentiation of isolates in the form of numerical codes reliably stored in databases, henceforth, we appreciate that MLVA would be used as a reasonable proxy for MLST. A good correspondence between MLST and MLVA was highlighted in previous studies. Therefore the MLVA scheme was accurate in identifying the population structure of *P. aeruginosa* [54].

### Phylogenetic relatedness within closely related isolates exemplified with CC235

The prominent clonal lineage detected in our collection was MLST-CC235. To gain additional resolution of the molecular evolution and dissemination of such epidemic clones, we analyzed this clonal complex separately. The 44 isolates belonging to this lineage were shown to be heterogeneous by PFGE (19 types), followed by MLVA (13 MTs), DL (9 types), and finally MLST (5 STs). This finding highlighted the “violation” of ST assignment of CC235 isolates similar to the observation of Deplano et al. [41] when analyzing the epidemiological concordance of ST235 isolates. This study revealed that ST235 isolates were split into diverse DL types. These results clearly shed light on the features of each typing technique and their relative capacities to discriminate and differentiate among isolates belonging to the same lineage. Importantly, this lineage is characterized by a great complexity reflected by micro-evolutionary events visualized by multiple patterns resolved by each technique.

The international spread of the CC235 isolates has been reported on several occasions [45], [73]–[82]. In fact this is an important successful clone disseminated over a long period and eminently associated with serotype O11, presence of the *exoU* virulence gene, and characterized by frequently being multidrug-resistant. Likewise, Harris and coworkers [83] have analyzed the population structure of a prominent lineage of *Staphylococcus aureus*, MRSA ST238, which has shown an intercontinental spread. By conducting a high throughput genomic approach based incisively on the SNPs that reside in the core genome, it was possible to trace an ideal phylogeny permitting to interrogate the geographical origin and intra-hospital spread of this pathogenic clone. However, MLST seems less suitable when trying to distinguish various clonally related isolates i.e. the *P. aeruginosa* CC235 isolates, *Escherichia coli* ST131 [84], methicillin-resistant *S. aureus* [85] and particularly monomorphic pathogens [32]. The limitation of MLST here is referred to the crude estimates of SNPs restricted within the housekeeping genes. The rapid evolution of *P. aeruginosa* clones such as the CC235 clone might decrease their genetic relatedness and obscure their epidemiological linkage, and eventually conflict the phylogenies extracted simultaneously by several techniques.

## Conclusions

In this study, we have compared the properties of four genotyping schemes for the phylogenetic analysis of *P. aeruginosa*. Although MLST is highly informative, it has a limited resolution, especially when applied to closely related isolates as shown for the prominent lineage CC235. MLVA was able to predict the MLST-type with high accuracy, and even higher at the clonal complex level, making it the best surrogate method for MLST in phylogenetic studies. Also, MLVA, as a relaxed scheme, provides sufficient resolution for strain differentiation and clustering analysis, and could also be used for outbreak investigation. The low throughput PFGE and the automated DL are useful for outbreak investigation, but to a lesser degree for long-term epidemiological studies. They are efficient to dissect subtle genomic differences especially among very closely related isolates.

## Supporting Information

Table S1 **Database displaying the genotypes retrieved by each genotyping schemes: MLST (STs), MLST-CC, MLVA (MTs), MLVA-CC, PFGE (cut-off 80%) and DL (cut-off 95%).**
(XLSX)

Table S2 **Index of diversity of each VNTR locus.**
(XLSX)
