# Correction: Four Genotyping Schemes for Phylogenetic Analysis of *Pseudomonas aeruginosa*: Comparison of Their Congruence with Multi-Locus Sequence Typing

**DOI:** 10.1371/journal.pone.0137931

**Published:** 2015-09-03

**Authors:** The PLOS ONE Staff

Tables 1 and 2 are incorrectly switched. The table that appears as Table 1 should be Table 2, and the table that appears as Table 2 should be Table 1. The table titles appear in the correct order. Please see the correct Tables 1 and 2 here. The publisher apologizes for these errors.

**Table 1:** Number of genotypes, clonal complexes and singletons retrieved from the four genotyping schemes.

Typing methods	MLST	MLVA	PFGE	DiversiLab
Genotypes/types	67 STs	85 MTs	90 PFGE-types	61 DL-types
Clonal complexes/clonal groups	11	11	20	25
Singletons/unique patterns	39	49	70	36

**Table 2:** Simpson's index of diversity of the different typing methods.

Typing methods	No. of types	SID	95% CI
**PFGE**	90	0.989	(0.984–0.995)
**MLVA**	85	0.980	(0.970–0.991)
**DL (cut-off 95%)**	61	0.961	(0.946–0.977)
**DL (cut-off 93%)**	46	0.937	(0.915–0.958)
**MLST**	67	0.906	(0.860–0.951)
